# Huangqin-Tang and Ingredients in Modulating the Pathogenesis of Ulcerative Colitis

**DOI:** 10.1155/2017/7016468

**Published:** 2017-06-12

**Authors:** Chunyan Wang, Xudong Tang, Li Zhang

**Affiliations:** ^1^Institute of Digestive Diseases, China-Canada Center of Research for Digestive Diseases (ccCRDD), Longhua Hospital, Shanghai University of Traditional Chinese Medicine, Shanghai 200032, China; ^2^Xiyuan Hospital of China Academy of Chinese Medical Sciences, Beijing 100091, China

## Abstract

Ulcerative colitis (UC) is the most common inflammatory bowel disease worldwide. Current therapies in UC cause limitations, and herb medicine provides an important choice for UC treatment. Huangqin-Tang (HQT) is a well-known classical traditional Chinese herbal formula and has been used in China for thousands of years. A large number of pharmacological studies demonstrated HQT and its ingredients to be effective in treating UC. Though the therapeutic effect has been evaluated, comprehensive up-to-date reviews in this field are not yet available. Here we aim to review our current understanding of HQT and its ingredients in treating UC and how the agents modulate the main pathogenesis of the disease, including the intestinal environment, immune imbalance, inflammatory pathways, and oxidative stress. The summary on this issue may provide better understanding of HQT and its ingredients in treating UC and possibly help in promoting its clinical application.

## 1. Introduction

Ulcerative colitis (UC) is the most common form of inflammatory bowel disease, it is characterized by chronic inflammatory disorders of the colonic mucosa, which starts in the rectum and generally extends proximally in a continuous manner through part of or the entire colon [1]. The disease is bifurcated into remitting and relapsing courses [2] and may have significant impact on the quality of life and personal burden through reduction in the ability to work [3, 4]. UC patients may require life-long treatment and have an increased risk of developing colorectal cancer [5, 6].

The global prevalence of UC is about 8 million, and the incidence and prevalence are increasing worldwide [7]. While the precise cause of UC is still unknown, it has been hypothesized that various factors such as geography, age, sex, genetic, environmental, gastrointestinal infection, and appendicectomy are responsible for the development of UC [1, 8–11]. The pathophysiology is related to epithelial barrier impairment, commensal microflora disorders, antigen recognition, dysregulation of immunological responses, leucocyte recruitment, and so forth. However, since the exact mechanisms of UC are still under investigation, treatment strategies are limited. The principle for UC treatment is divided into two categories according to the clinical activity and the extent of diseases, induction of remission, and maintenance of remission. The main agents consist of topical or oral mesalazine, oral or intravenous corticosteroids, immunosuppressive drugs, monoclonal antibodies of TNF-*α*, and colectomy [1]. However, the side effects and high cost limit the long-term application [12].

Recently, natural products have attracted lots of interest in preventing and treating UC. Traditional Chinese Medicines (TCM) and extracts have shown various beneficial treatment effects including bacteriostasis, anti-inflammation, and anticancer abilities [13]. Among them, Huangqin-Tang (HQT) is a well-known classical formula which derived from Shang Han Lun, consisting of four ingredients: the roots of* Scutellaria baicalensis *Georgi (scute),* Glycyrrhiza uralensis *Fisch (licorice),* Paeonia lactiflora *Pall. (peony), and the fruit of* Ziziphus jujuba *Mill (Chinese date). Using high-quality herbs picked by experienced herbalists and manufactured according to cGMP (current Good Manufacturing Practice), HQT was extracted and named as PHY906 [14]. Using standardized chemical and biological fingerprints, the consistent preparations of PHY906 have been made and developed as an adjuvant for chemotherapy in various cancers.

**Figure 1:**
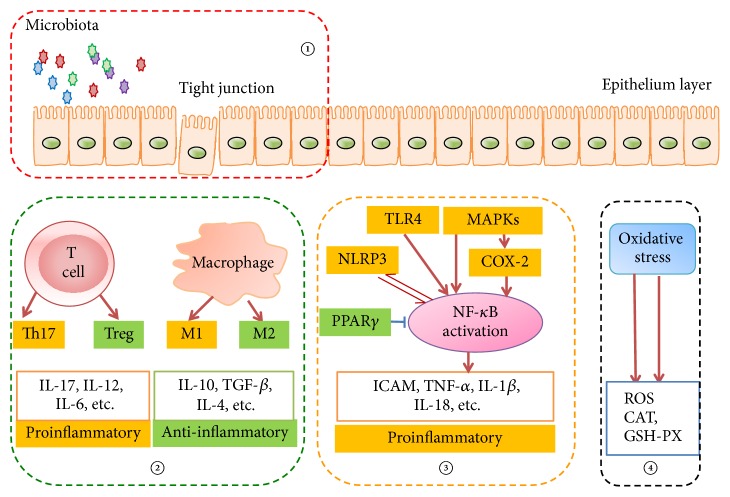
*HQT regulates the main pathogenesis of UC*. ① HQT and its ingredients modulate intestinal microbiota and the integrity of epithelium; ② HQT and its ingredients maintain the immune balance (Th17 versus Treg; M1 versus M2 type macrophages); ③ HQT and its ingredients target the inflammatory pathways, which could result in decrease of proinflammatory cytokines release; ④ HQT and its ingredients improve oxidative stress via enhancing antioxidants production.* Note*. ↑ indicates activated action and T indicates inhibited action.

Though HQT has been widely used in treating UC in China, the underlying mechanisms are still not clear. Pharmacokinetic studies on multiconstituents in HQT by the validated HPLC method showed that the main ingredients of HQT included baicalin, wogonoside, oroxylin-A-glucuronide, baicalein, wogonin, oroxylin-A, paeoniflorin, paeonimetabolin-I, liquiritin, liquiritigenin, glycyrrhizic acid, glycyrrhetinic acid, and visidulin I [15]. These constituents can have anti-inflammatory effect through different or similar mechanisms in UC treatment. Application of HQT or its ingredients in UC has been widely investigated; however, the systematic effect evaluation and mechanisms exploration are spotted, and we aim to summarize the current studies about the therapeutic effects or mechanisms of HQT or its main ingredients in UC (Figure 1), thus to better support its clinical use.

## 2. HQT and Ingredients in Targeting Intestinal Environment on UC

Imbalanced interactions with intestinal microbes lead to development of UC, and intestinal microbiota and epithelium provide environment for the pathogens.

There are approximately 10^11^~10^14^ enteric commensal microorganisms in the gut. Under normal conditions, these commensal bacteria maintain the balance and help in regulating crucial nutrient provision, immune response, and energy metabolism. However, commensal microorganisms can be noxious for intestinal inflammation under certain circumstances, and the diversity and amount of microbiota are reduced in patients with UC compared to that in healthy humans [14]. Though the specific bacteria related to the incidence of UC have not been found, lots of studies have showed that the change of intestinal environment was closely related to UC, and there was significantly different bacterial colonies between UC patients and healthy people [16–18]. PHY906, the extracted HQT, has been reported to alter the profile of major intestinal bacteria species, and treatment of mice with PHY906 could significantly decrease* Bacteroides* and* E. rectale/C. coccoides *and increase* Clostridium leptum* specially in the colon [19]. In addition,* Paeonia lactiflora *root, the main constituent of HQT, has been demonstrated to inhibit the growth of harmful intestinal bacteria in human [20].

The intestinal epithelial cells structurally constitute crypts and villi in the intestine, with a single columnar cell lining with a tight junction. The epithelial integrity is maintained by tight junctions between epithelial cells, and external pathogens easily pass through the injured or incomplete intestinal epithelium, which is known to be involved in the pathogenesis of UC. Several lines of evidence showed that the ingredients of HQT can protect the epithelium from pathogen introduction. Wogonin is a flavonoid isolated from* Scutellaria baicalensis *Georgi, the predominant ingredient of HQT. Pretreatment of Caco-2 cells with wogonin protects intestinal barrier function in lipopolysaccharide (LPS) stressed condition. Both 10 *μ*M and 50 *μ*M wogonin attenuate the LPS-induced transepithelial electrical resistance and transport of fluorescent markers and upregulates claudin-1 and ZO-1, the representative tight junction proteins in intestine [21]. Paeoniflorin, the main active ingredient of* Paeonia lactiflora *Pall., is confirmed to attenuate LPS-induced permeability in endothelial cells, and the ingredient (10, 30, and 100 *μ*M) could inhibit dextran extravasation and leukocyte migration in a concentration-dependent manner [22]. Furthermore, the ingredient glycyrrhetinic acid also exerted protective effects on LPS stressed intestinal epithelial cells injury and expression of the epithelial tight junction molecules [23].

## 3. HQT and Ingredients in Regulating Immune Imbalance

The gut possesses an abundant and highly active immune system that is tightly regulated to prevent overreaction of immune responses [24]. Studies have provided the evidences that the dysfunction of gut immune system, mainly involving the abnormal percentage and disturbed differentiation of immune cells, can result in the increase of proinflammatory cytokines, epithelial permeability, and subsequent intestinal mucosa damage [25–27]. Both Th17 and regulatory T (Treg) cells are originated from CD4^+^ T cells. Th17 cells, which secrete IL-17 and IL-22, promote inflammation process. On the contrary, Treg cells, which produces IL-10 and transforming growth factor-*β* (TGF*β*), inhibit the inflammatory response [28, 29]. As one important subtype of T cells, CD4^+^CD25^+^ forkhead box p3^+^ (Foxp3^+^) Treg cells inhibit autoimmunity and protect the tissue against injury and their development is controlled by Foxp3.

Growing evidences suggest that the imbalance between Th17 cells and Treg cells may contribute to the development of UC [30–32]. The ingredients of HQT are reported to regulate immune imbalance in a series studies. In trinitrobenzenesulfonic acid- (TNBS-) induced UC mice, baicalin could ameliorate the severity of the disease by downregulating the number of Th17 cells and the levels of Th17-related cytokines (IL-17 and IL-6) and upregulating Treg cells and related TGF*β*, IL-10, and Foxp3 levels [33]. Similar effect of baicalin was also observed in cultured T cells; it proved that baicalin induced Foxp3 protein expression and inhibited T cells proliferation [34]. CD4^+^CD29^+^ cells are helper T cells that can result in high activation of B cells and abnormal immune response. Previous study showed the percentage of CD4^+^CD29^+^ T cells significantly increased in UC patients, indicating CD4^+^CD29^+^ T cells might be an immunology index for monitoring UC [35, 36]. Baicalin can reduce the percentages of CD4^+^CD29^+^ T cells in cultured peripheral blood mononuclear cells from the UC patients, thus regulate immune balance, and relieve the UC-induced inflammation [37]. These studies inferred that baicalin may serve as a promising natural immunosuppressive compound for treating UC and related inflammatory diseases.

Macrophages play critical roles in both innate and adaptive immune responses and are classified into M1 and M2 phenotypes. M1 phenotype cells are stimulated by microbial products or other pathogens and produce many proinflammatory cytokines. In contrast, M2 type responses are the “resting” phenotype and are observed in healing-type circumstances without infections and generate anti-inflammatory cytokine. Macrophage M1 and M2 types execute opposite activities in tissues [38]. Once the M1/M2 balance is broken, immune-mediated inflammation occurs. Baicalin is reported to polarize macrophages to an M2 phenotype in murine peritoneum and ameliorate experimental inflammatory bowel disease in mice [39]. In mouse bone marrow precursors generated M1 and M2 cells, paeoniflorin inhibited LPS-induced M1 activity by reducing iNOS and NO production, whereas enhanced IL-4 provoked M2 function by upregulating Arg-1 production and activity [40], suggesting paeoniflorin can suppress M1 cells activity and enhance M2 cells function simultaneously. In addition, paeoniflorin is confirmed to significantly ameliorate the immune complex induced vascular damage, leucocyte infiltrates, and adhesion molecules expression [41]. The pharmaceutical effect of baicalin is reported to be associated with macrophage migration inhibitory factor downregulation, the quantity of macrophages, and the amount of M macrophage-related cytokines and macrophage inflammatory protein-3*α* [42].

B cells are important in the development of autoimmune disorders. Autoreactive B cells can present self-antigens to autoreactive T cells, produce autoantibodies and proinflammatory cytokines, and amplify inflammatory responses [25, 43, 44]. Inhibiting the abnormal activation of B cells is thought to be an effective strategy for UC treatment. It is reported that the B cell activation can be inhibited by paeoniflorin; in LPS-stimulated murine spleen B cells, paeoniflorin inhibits CD69/CD86 expression and B cell proliferation [45].

## 4. HQT and Ingredients in Modulating Inflammatory Pathways

Interactions between proinflammatory cytokines and their receptors lead to activation of intracellular signal transduction. And NF-*κ*B has been recognized as a critical target in inflammatory process. The extracellular stimulus, like Ag, LPS, growth factors and inflammatory cytokines, signals, and so forth, could trigger NF-*κ*B activation and induce inflammation in tissues. Therefore, inhibited NF-*κ*B activation is considered to be an effective strategy for UC treatment [46].

Toll-like receptor 4 (TLR4), a key receptor for commensal recognition in innate immunity, is overexpressed in inflamed colonocytes [47, 48], and TLR4-mediated signal transduction can evenly lead to NF-*κ*B activation [49]. In LPS stressed RAW264.7 cells, baicalin can block the TLR4/NF-*κ*B pathway and inhibit IL-6 release and cell proliferation; in Sprague-Dawley rats, TNBS-induced UC status can be ameliorated by baicalin [50, 51]. In DSS-induced C57BL/6 mice, paeoniflorin could improve UC, and the beneficial effects are considered to be related to the downregulation of TLR4 expression and the blockage of NF-*κ*B activation [52]. In addition, wogonin could attenuate the TLR4-mediated inflammatory response and maintain the single-layer membrane structure under LPS stressed Caco2 cells, and the positive effects might be achieved via TLR4-MyD88-TAK1-mediated NF-*κ*B pathway [21].

Mitogen-activated protein kinases (MAPKs) are conserved among all eukaryotes and participate in multiple cellular processes including cell growth, proliferation, differentiation, migration, inflammation, and survival. Numerous studies have described an increased expression of MAPKs in IBD patients [53]. There are several studies focusing on the therapeutic effects for UC by regulating MAPKs. Glycyrrhetinic acid had the inhibitory effects on IL-8 production in intestinal epithelial cells through blocking MAPKs phosphorylation, followed by I-KB*α* degradation and NF-*κ*B activation [54]. Similarly, isoLQ, a chalcone found in licorice, could attenuate the DSS-induced colitis via suppressing MAPKs phosphorylation and NF-*κ*B activation in inflamed colon tissue [55]. COX-2, one isoform of cyclooxygenase (COX), can be inducted by activating MAPK activity [56, 57]. And previous research has shown that the level of COX-2 increased in the intestinal tissues of UC, and COX-2 inhibitor could relieve intestinal inflammation in rats with UC [58–60]. PF2405, a standardized fraction of* S. baicalensis*, can significantly inhibit TNF-*α* induced COX-2 expression through JNK1/2 dephosphorylation and p38 MAPK in HT-29 cells and reduced the expression of proinflammatory cytokines and COX-2 in TNBS-induced colitis in female C57BL/6 mice [61].

Intercellular adhesion molecule 1 (ICAM-1) and TNF-*α* are the representative cytokines in inflammatory injuries. ICAM-1 induces the migration and infiltration of inflammatory cells into the lesion, whereas TNF-*α* can alter vascular and intestinal permeability [62]. It is reported that diammonium glycyrrhizinate could reduce TNF-*α* and ICAM-1 by inhibiting the NF-*κ*B activation and improve intestinal inflammatory injury in a rat model [63]. NLRP3 inflammasome is a key component of inflammatory process and its dysregulation contributes to UC. The synthesis and accumulation of NLRP3 inflammasome can be activated by NF-*κ*B and lead to the secretion of IL-1*β* and IL-18. It is reported that WG (the glucuronide metabolite of wogonin) could ameliorate DSS-induced colitis via inhibiting NF-*κ*B and NLRP3 inflammasome activation [64]. In addition, there are studies that focus on the inflammatory cytokines. Paeoniflorin can play anti-inflammatory action by increasing the level of IL-17 and decreasing the level of IL-10 in recombinant human IL-1*β*-stimulated human peripheral blood mononuclear cells in vitro [65]. The glycyrrhizic acid could significantly reduce TNF-*α* and IL-1*β* levels in TNBS-induced colitis rats, and in vitro experiments indicated that glycyrrhizic acid could inhibit IL-6 and elevate IL-10 production in LPS-activated macrophages and significantly inhibit lymphocytes proliferation [66]. And similar effects are obtained in DSS-induced inflamed mucosa in rats [67].

Peroxisome proliferator-activated receptor *γ* (PPAR*γ*), a member of the nuclear receptor family, has been recognized as an endogenous regulator of intestinal inflammation. It is reported that activating PPAR*γ* can inhibit NF-*κ*B activation. Oroxyloside, one of the ingredients of HQT, has been reported to activate PPAR*γ* and prevent DSS-induced colitis through inhibiting NF-*κ*B pathway [68]. Moreover, thioredoxin system is implicated in the regulation of NF-*κ*B transactivation potential; there is one study that focuses on the inhibition of baicalin on the thioredoxin system and finds that baicalein can suppress mitogen induced thioredoxin activity in the unclear compartment of lymphocytes, thus limiting NF-*κ*B dependent inflammatory responses [69]. In addition, it has been reported that estrogen can exert anti-inflammatory effects. Based on this background, one study shows that baicalein has estrogen-like activity and inhibits LPS-induced inflammatory cytokine production via regulating NF-*κ*B pathway, suggesting the potential efficacy in preventing inflammation related diseases [70]. Scutellariae Radix extract was effective in treating acute DSS-induced UC with improving macroscopic and histological damage scores and enhancing recovery of normal colonic secretory function [71].

## 5. HQT Ingredients in Inhibiting Oxidative Stress

The available evidence suggests that oxidative stress may be involved in the pathogenesis of UC [72, 73]. The reactive oxygen species (ROS) are produced in excess by the inflamed mucosa and overwhelm the endogenous defenses in inflammatory intestinal diseases [74]. Among the masses of related products of oxidative stress, catalase (CAT) and phospholipid hydroperoxide glutathione peroxidase (GSH-Px) can reduce the intestinal damage by strengthening the oxidation resistance or reducing lipid hydroperoxides to their corresponding alcohols [75, 76]. One study showed that the possible mechanisms of baicalin in protecting UC were associated with the inhibition of oxidative stress; baicalin could increase the activities of CAT and GSH-Px in LPS-stimulated RAW264.7 cells and TNBS-induced UC rats [77].

## 6. Conclusions and Perspectives

In summary, available reports suggested that the mechanisms under the efficacy of HQT or its components on UC are related to intestinal environment improvement, immune modulation, and regulation of inflammatory pathways or cytokines (Figure 1). Since most data are interpreted from animal studies or in vitro experiments, the effects and mechanisms of HQT in UC patients remain to be explored or verified. In addition, most of the current studies are observational studies and the targets of the components remain unclear.

Though, along with the upgrade of the Chinese drugs ingredient detection tools, the compounds of Chinese herbal prescription have made some progress, its standardization is still the important obstacle for traditional Chinese medicine. And these studies are influenced by the quality, origin, and different processing method of single herb. Future studies should focus on the standardization of the compounds and the best compatibility in accordance with the disease database and traditional Chinese medicine database in order to achieve the best effect. In addition, different prescriptions for the acute phase or remission phase of UC patients should also be considered.
